# Artificial Intelligence Application to Screen Abdominal Aortic Aneurysm Using Computed tomography Angiography

**DOI:** 10.1007/s10278-023-00866-1

**Published:** 2023-07-05

**Authors:** Giovanni Spinella, Alice Fantazzini, Alice Finotello, Elena Vincenzi, Gian Antonio Boschetti, Francesca Brutti, Marco Magliocco, Bianca Pane, Curzio Basso, Michele Conti

**Affiliations:** 1 Department of Surgical Sciences and Integrated Diagnostics (DISC), University of Genoa, Viale Benedetto XV 6, 16132 Genoa, Italy; 2 Vascular and Endovascular Surgery Clinic, IRCCS Ospedale Policlinico San Martino, Largo R. Benzi 10, 16132 Genoa, Italy; 3 Camelot Biomedical System, Genoa, Italy; 4 IRCCS MultiMedica, Milan, Italy; 5 Department of Computer Science, Robotics and Systems Engineering, University of Genoa, BioengineeringGenoa, Italy; 6 Vascular Surgery Unit, AULSS 2 Marca Trevigiana, Treviso Hospital, Treviso, Italy; 7 Department of Mathematics, University of Trento, Trento, Italy; 8 Department of Civil Engineering and Architecture, University of Pavia, Pavia, Italy

**Keywords:** Artificial intelligence (AI), Deep learning (DL), Abdominal aortic aneurysm (AAA), Screening

## Abstract

The aim of our study is to validate a totally automated deep learning (DL)-based segmentation pipeline to screen abdominal aortic aneurysms (AAA) in computed tomography angiography (CTA) scans. We retrospectively evaluated 73 thoraco-abdominal CTAs (48 AAA and 25 control CTA) by means of a DL-based segmentation pipeline built on a 2.5D convolutional neural network (CNN) architecture to segment lumen and thrombus of the aorta. The maximum aortic diameter of the abdominal tract was compared using a threshold value (30 mm). Blinded manual measurements from a radiologist were done in order to create a true comparison. The screening pipeline was tested on 48 patients with aneurysm and 25 without aneurysm. The average diameter manually measured was 51.1 ± 14.4 mm for patients with aneurysms and 21.7 ± 3.6 mm for patients without aneurysms. The pipeline correctly classified 47 AAA out of 48 and 24 control patients out of 25 with 97% accuracy, 98% sensitivity, and 96% specificity. The automated pipeline of aneurysm measurements in the abdominal tract reported a median error with regard to the maximum abdominal diameter measurement of 1.3 mm. Our approach allowed for the maximum diameter of 51.2 ± 14.3 mm in patients with aneurysm and 22.0 ± 4.0 mm in patients without an aneurysm. The DL-based screening for AAA is a feasible and accurate method, calling for further validation using a larger pool of diagnostic images towards its clinical use.

## Introduction

The abdominal aortic aneurysm (AAA) is a well-recognized life-threatening disease, although it may often be asymptomatic [1, 2]. An aneurysm is a permanent and localized dilation of an artery having at least a 1.5 times larger in diameter compared with the adjacent normal segment and commonly defined as greater than 30 mm for AAA [3]. Abdominal aortic aneurysm represents a relevant public health problem with a prevalence between 1.3 and 12.5% in males and between 0.0 and 5.2% in females [4]. Given the frequent inflammatory component of aneurysmal degeneration, an atherosclerotic-driven intraluminal thrombus covers the vessel wall in 70–80% of AAA [5].

Screening programs are essential tools that have proven to be effective in AAA detection and can reduce specific and overall mortality, especially in the male population [6–11]. In fact, AAA screening in women has long been a matter of debate despite being currently indicated in an at-risk population and suggesting the aneurysm diameter indexed to body size as a predictor of rupture [2].

International guidelines recommend screening programs be performed with Doppler ultrasound (DUS) [1, 2]. However, screening programs are a time-consuming activity that require many resources, and not all healthcare systems have a policy to establish it for AAA detection. Given the widespread use of computed tomography (CT) as the method of choice for the diagnosis of many pathologies, it is not uncommon to incidentally find an AAA (from 1 to ~ 6% depending on the patient’s cohort), even if they are not always reported [12–14]. Although this incidence differs when compared to those found in a dedicated screening program, the incidental findings are relevant with regards to properly monitoring and electively repairing AAAs.

In this context, automated analysis tools may improve these aspects, both allowing for the analysis of a large amount of data produced by the extensive use of CT scans in clinical practice and also by avoiding the time consuming process of diagnostic images reporting. In the last few years, several software analyzing aortic anatomies have been developed to support experts in clinical practice [15–17]. However, the majority of this software is semi-automated and focused on the analysis of the aortic lumen, without considering any intra-luminal thrombi and thus not measuring the maximum diameter of the aneurysm. Artificial intelligence (AI) and deep learning (DL) have been recently applied to automatically evaluate aortic morphology and diameters on CT angiographies (CTA) which helps physicians better characterize AAAs by predicting their evolution and postoperative complications [18–23]. Those concepts could be summarized by the ability of a program to imitate intelligent human behavior (AI methods) and a subset of algorithms in which multilayered neural networks learn from a huge amount of data (DL). For example, Adam et al. created an automated AI algorithm that can assess the maximal diameter of aortic aneurysm both before and after surgery [20], while Lareyre et al. automatically measured the AAA diameter growth over time [22]. Moreover, Caradu et al. tested and compared the accuracy of AI-based AAA segmentation with manual segmentation [23].

The application of these tools into screening programs is a novel concept of high interest due to its implications in early disease detection and therapeutic pathway creation [24–26]. In detail, Golla et al. created an automated screening method for AAA using DL in CT scans. The system underwent clinical testing, and the findings were encouraging [24]. The use of natural language processing to determine the existence and extent of AAA was validated by McLenon et al. The technique demonstrated good accuracy and might be helpful in AAA screening programs [25]. Camara et al. created an AAA detection convolutional neural network (CNN). The CNN demonstrated good specificity and sensitivity, and it may boost the accuracy of AAA screening [26].

In our previous works, we have developed an automatic pipeline for the identification and segmentation of the aortic lumen from CTA images [27] and a methodology to automatically segment the abdominal thrombus and automatically measure the AAA diameter [28]. These studies set the ground for the present work that proposes a novel methodology for measuring AAA diameter.

In this scenario, in order to perform automatic AAA screening, we proposed a pipeline based on automatic lumen and thrombus segmentation and maximum abdominal aneurysm diameter calculation.

## Methods

### Study Design

This retrospective study is based on a dataset of 73 thoraco-abdominal CTA images performed between 2014 and 2020. The mean age was 75 ± 8 years, and 55 patients (76%) were male. These CTAs were performed in six different institutions without a standardized acquisition protocol and with 11 different scanners, belonging to SIEMENS, PHILIPS, and GE Medical Systems manufacturers (Table 1). Each CTA image was manually analyzed by an expert vascular surgeon with more than 10 years of experience who detected and manually measured the maximum diameter. The manual measurement of the maximum diameter (aortic lumen and parietal thrombus) is performed for each patient using commercial software for advanced planning and sizing of the aorta (EndoSize version 3.1.47 Therenva SAS, Rennes, France). In particular, the manual measurements are performed considering the maximum diameter of the infrarenal aorta (wall to wall) perpendicular to the centerline. These measurements are blinded to those collected by automatic pipeline. In case the measured diameter is larger than the threshold value (30 mm), the CTA image is labeled as AAA, otherwise as control. Specifically, 66% (*n*, 48) of them referred to patients labeled by radiologists as “aneurysmatic,” while 34% (*n*, 25) were used as controls. Thus, in the AAA analysis step, the ground truth is represented by the manual measurements performed by an expert operator in the abdominal tract.

**Table 1:** Description of the dataset adopted to validate the automatic pipeline proposed to perform automatic abdominal aortic aneurysm screening

**Patient demographics**	
Sex, *n* (%)	Male = 55 (76%) Female = 18 (24%)
Age, years ± sd	75 ± 8
**CT information**	
Scans, *n*	73
Manufacturers	GE MEDICAL SYSTEMS
	Light Speed Pro 32 Light Speed VCT Light Speed 16 Optima CT660 Revolution CT
	SIEMENS
	Somatom Definition AS Somatom Definition AS + Somatom Definition Flash Sensation 40 Sensation 64
	**PHILIPS**
	Ingenuity CT
Pixel spacing (*x*,* y* axis), mm ± sd	0.74 ± 0.11
Slice thickness (*z* axis), mm ± sd	0.75 ± 0.30
**Institutions**	
Number of institutions, *n*	6
**Aortic characteristics**	
AAA, *n* (%)	48 (66%)
AAA max diameter, mm ± sd	51.1 ± 14.4
AAA diameter, mm range [min, max]	[30.1, 104.6]
No AAA, *n* (%)	25 (34%)
No AAA max diameter, mm ± sd	21.7 ± 3.6
No AAA, diameter range [min, max]	[15.7, 29.3] mm
Full dataset (AAA, no AAA), max diameter	41.3 ± 18.4 mm
Full dataset (AAA, no AAA), diameter range [min, max]	[15.7, 104.6] mm

*AAA* Abdominal aortic aneurysms, *CT* Computed tomography

The study was approved by the Independent Ethics Committee of Regione Liguria (2021/451), all patients gave signed consent to the processing of personal and clinical data, and all the procedures were performed in accordance with the Declaration of Helsinki.

### Study Methodology

#### Proposed Pipeline

The DL pipeline designed and previously validated by Fantazzini et al. and Brutti et al. was adopted to automatically segment the aortic lumen and intraluminal thrombus from CTAs [27, 28]. These publications provide the basis for the work that is presented in this paper in which we propose a new methodology for measuring AAA diameter. Therefore, their main features are briefly summarized below in order to emphasize what is new in the current work compared to the work previously published by our group. Regarding automatic lumen and thrombus segmentation, our developed pipeline exploits a 2.5D approach and combines three single-view CNNs with a final multi-view integration phase [27, 28]. Compared to simple 2D CNNs, our segmentation approach allows spatial context to be considered. Moreover, in comparison with 3D CNNs, our pipeline requires less data for training and can take advantage of more complex architectures.

Regarding the pipeline developed to automatically segment and analyze abdominal thrombus [28], in the developed procedure, both lumen and thrombus are automatically segmented from CTA scans, and then, the spatial extent of the thrombus segmentation is used to mask the segmentation of the lumen, which extends from the aortic root to the common iliac arteries. To analyze only the abdominal aneurysm and exclude healthy sections of the aortic lumen, the lumen segmentation is cropped considering the first and last slices into which the thrombus is segmented. At this point, the centerline of the lumen is extracted, and thus, the aneurysm sections can be evaluated to extract quantitative measures. Therefore, the pipeline developed in our previous work [28] depends entirely on thrombus identification and segmentation. In particular, note that if the thrombus is not identified in the CTA scans, no geometric analysis is performed; conversely, if the thrombus is identified, there is no information about its location in the analysis (thoracic, abdominal, or iliac area). In fact, although the thrombus segmentation model was trained only on abdominal thrombi, this model is able to generalize and identify thrombi not necessarily in the abdominal area.

Therefore, to perform automated screening of abdominal aneurysms, the previously developed pipeline [28] needs to be modified and extended. First, it is necessary to automate the extraction of the centerline of the whole aorta (aortic lumen and thrombus, if present) so that it is decoupled from the segmentation of the thrombus, thus allowing the diameters to be measured even if the thrombus is not present. Second, the analysis must be focused on a specific tract of the aortic anatomy that is the abdominal tract. In fact, for the abdominal aorta, diameters considered to be aneurysmal have different measurements from thoracic ones (30 mm for abdominal, 45 mm for thoracic). Thus, correctly identifying the area of interest is crucial in order to compare the extracted diameters with the corresponding clinical threshold (in our case, the abdominal diameter is compared with the 30-mm threshold).

Thus, the work proposed in this paper exploits the automatic lumen and thrombus segmentation networks developed and validated in previous works [27, 28] but modifies and extends the geometric analysis aimed at screening abdominal aneurysms. More specifically, given the aorta and thrombus segmentation, the lumen centerline is extracted in the abdominal tract, and the diameters of the sections perpendicular to the centerline are automatically calculated. If the maximum calculated diameter is greater than the selected threshold diameter, the patient is defined as aneurysmal.

Each of the above-mentioned three main steps (automatic lumen and thrombus segmentation, automatic centerline extraction from lumen segmentation, and aortic diameter analysis) is described in detail in the following. No manual interaction is required in the whole pipeline.

#### Automatic Lumen and Thrombus Segmentation

In our previous works, we have developed an automatic pipeline for identification and segmentation of aortic lumen from CTA images [27] and a methodology to automatically segment the thrombus [28].

**Fig. 1:**
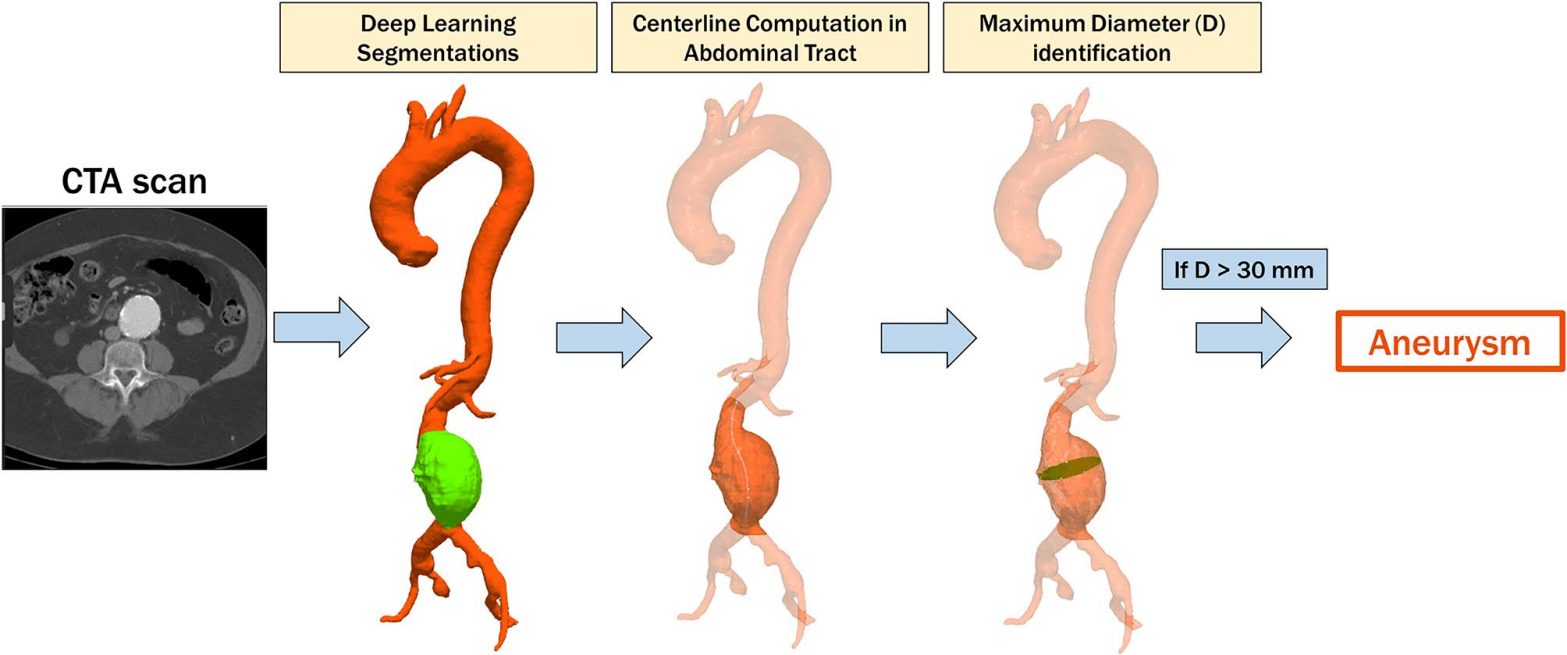
Pipeline proposed to perform automatic aneurysm screening. First, aortic lumen and thrombus are extracted from the CTA scan with a deep learning algorithm. Then, the lumen centerline is computed in the abdominal tract. Finally, the maximum abdominal aortic diameter is extracted. If the diameter is greater than the threshold value, the patient is considered “aneurysmatic”

**Fig. 2:**
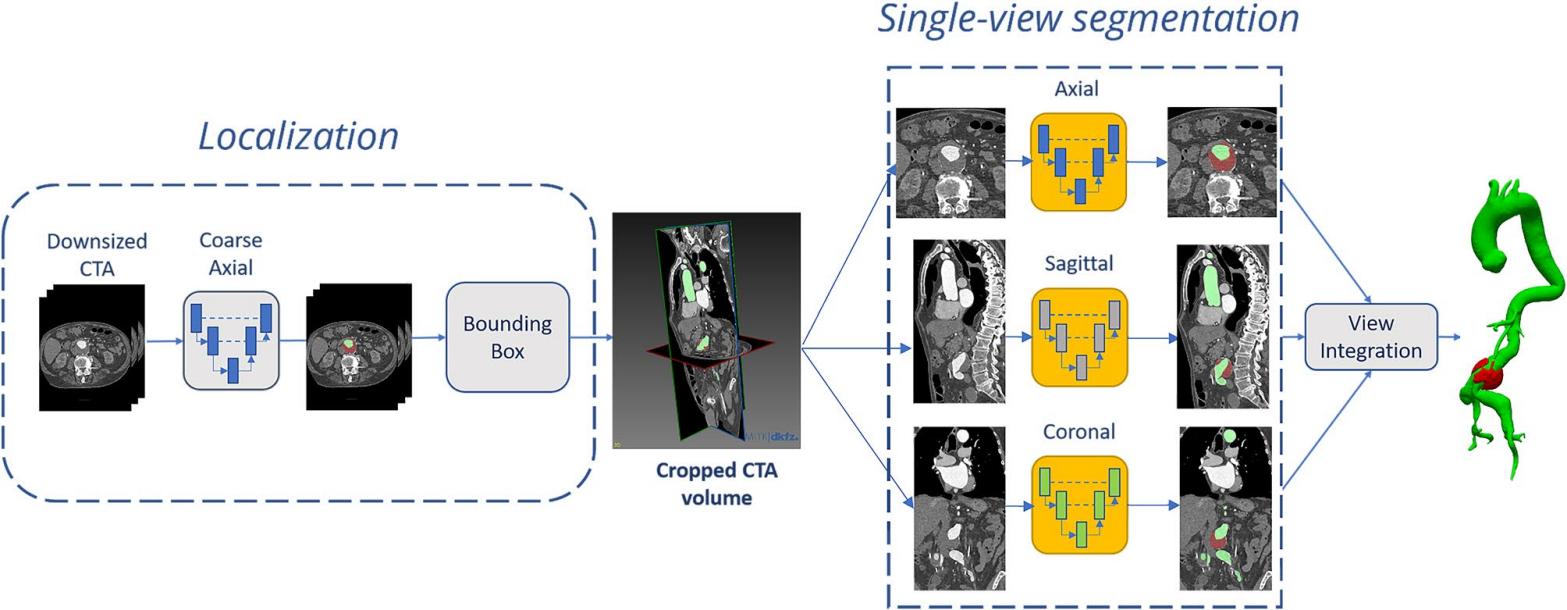
Pipeline performing automatic lumen segmentation from CTA images. A first network is used to localize the region of interest (ROI) containing the aorta (in green); then, the ROI is processed by three orthogonal networks. The predictions are integrated to obtain a final segmentation. The same pipeline is adopted to segment the intraluminal thrombus (in red) from CTA scans

The segmentation pipeline is consisted of a first 2D CNN with U-Net architecture [29] and roughly segments the images to identify the region of interest within the resampled Angio-TC dataset (Fig. 1). The U-Net is a CNN architecture developed for segmentation of images. The identified region of interest (ROI), containing the aortic lumen and thrombus, was then processed at a higher resolution by single-view U-Nets trained on the axial, sagittal, and coronal plane. The localization step reduces the computational load on the networks for single-view segmentation and consequently the time required, improving performance. Finally, the predictions provided by the orthogonal U-Nets were combined to provide a final 3D-segmentation that was spatially coherent (Fig. 2). As it has been explained in detail in publications on segmentation methods [27, 28], multi-view integration is performed using a simple averaging approach. Specifically, the final prediction map for each voxel *x* is derived by averaging the single-view prediction maps, as follows: ${p}_{\mathrm{final}}\left(x\right)=\frac{1}{3}\;{p}_{\mathrm{axial}}\left(x\right)+\frac{1}{3}\;{p}_{\mathrm{sagittal}}\left(x\right)+\frac{1}{3}\;{p}_{\mathrm{coronal}}\left(x\right)$. The adoption of a 2.5D approach enables to overcome the limitations of single 2D networks, which do not consider spatial coherence on the *z*-axis, and 3D networks, which are computationally and data demanding.

The Dice similarity coefficient (DSC) was calculated as twice the pixels in common between the two segmentations (predicted both by the network and manual) and divided by the union of the two segmentations. Therefore, the DSC could range between 0 (no overlap between ground truth and predicted segmentations) and 1 for a perfect overlap.

The results shown in Table 2 represent the segmentation performance obtained in our previously published articles. Specifically, the table is reported for the sole purpose of summarizing the performance obtained testing the models used for lumen and thrombus segmentation, while specifying how the partitioning of the data in training-validation-test sets was performed. Thus, the CTA scans reported are those that were used to train, validate, and test the segmentation models at the time of paper publication [27, 28], respectively. These already trained models are used to obtain estimated segmentations of aortic lumen and thrombus on new unseen CTA scans to validate the automatic screening pipeline proposed in this paper.

**Table 2:** Performance of the deep learning-based segmentation models previously published in [23, 24]. The CTA scans are parsed into axial, sagittal, and coronal images to train, validate, and test the corresponding single-view models used to segment the aortic lumen and thrombus. The DSC reported in this table is obtained by integrating the single-view segmentations

**Segmentation**	DSC	Number of training CTA scans	Number of validation CTA scans	Number of test CTA scans
Lumen	0.93 ± 0.02	64	6	10
Thrombus	0.89 ± 0.04	63	12	14

*CTA* Computed tomography angiography, *DSC* Dice similarity coefficient

**Fig. 3:**
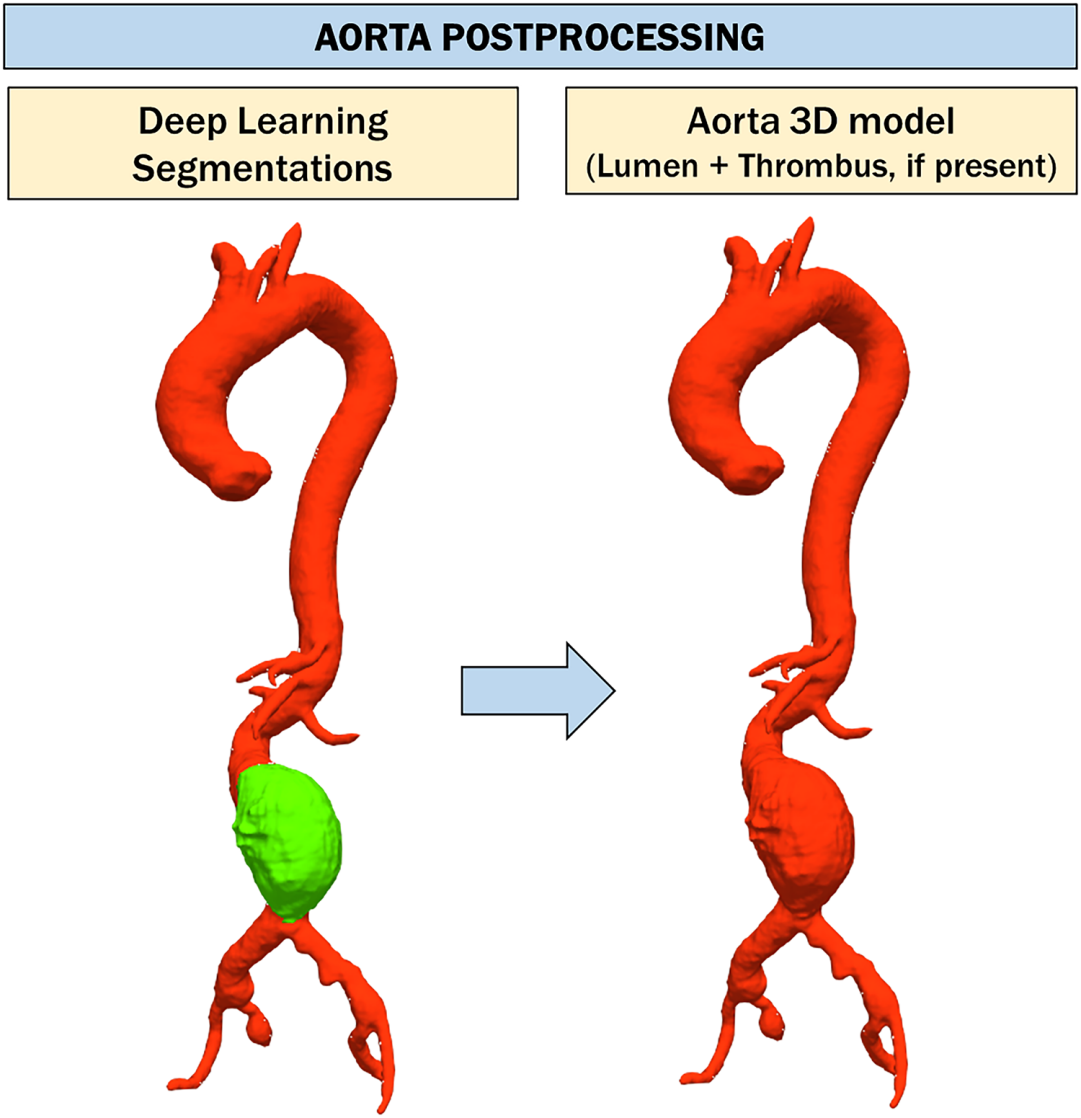
Aorta post-processing step. First, aortic segmentation is obtained by a summation operation between the segmentation of the aortic lumen and the thrombus, respectively. Then, this segmentation undergoes a smoothing process exploited to eliminate irregularities that can adversely affect the next steps. Finally, the polygonal model of aorta (lumen + thrombus, if present) is obtained using the marching cube algorithm [31]

Given the segmentations of the aorta and thrombus, the model of the entire aorta (consisting of lumen and thrombus, if present) is extracted (Fig. 3). The abdominal tract (between the origin of the superior renal artery and the iliac bifurcation) is automatically identified. Then, the central line of the aorta is automatically extracted and exploited to assess the maximum diameter of the aneurysm in the equidistant perpendicular sections identified along it. If a thrombus is not present, only the maximum diameter of the aortic lumen is calculated. As shown in Fig. 1, all analyses are limited to the abdominal area.

Since an abdominal tract with a diameter greater than 30 mm is considered an aortic aneurysm [3], the maximum diameter extracted with our pipeline was compared with this threshold value to determine whether the patient had an aneurysm or not: if the measured diameter was greater than the threshold value, the abdominal aorta was considered aneurysmal [30–32].

Starting from our segmentation pipeline, the aim of our study is to apply the methodological framework of AI and DL to screen AAAs in CT scans. No manual interaction is required in the whole pipeline.

To summarize, the following steps are introduced in the current geometric analysis pipeline:

1. Automatic centerline extraction from automatic whole aorta segmentation (aortic lumen + thrombus, if present), from the aortic arch to the iliac arteries
2. Automatic identification of the abdominal centerline, included between the renal and iliac arteries
3. Calculation of maximum abdominal diameter and comparison of calculated diameter with clinical threshold

#### Automatic Centerline Extraction from Automatic Whole Aorta Segmentation from the Aortic Arch to the Iliac Arteries

The lumen and thrombus segmentations provided by the deep learning networks are used as input to the screening pipeline. As a first step, aortic segmentation is obtained by a summation operation between the segmentation of the aortic lumen and the thrombus, respectively. Then, both lumen and aortic segmentation undergo a smoothing process exploited to eliminate irregularities that can adversely affect the next steps. More specifically, a process of dilation and erosion is adopted to make the surface of the segmented structures more regular. After smoothing, the polygonal model of the lumen and aorta (lumen + thrombus, if present) is obtained from the automatic segmentation using the marching cube algorithm [33].

Since the aortic segmentation includes thoracic, abdominal, and iliac aortic segments, additional steps must be taken to limit the analysis to the abdominal tract, between the origin of the superior renal artery and the iliac bifurcation. Through a process of connected component analysis performed slice by slice on the axial view, *z*-coordinates related to the aortic arch and iliac arteries are automatically identified. Then, the 3D models of lumen and aorta (lumen + thrombus, if present) are cropped between these two *z*-coordinates so as to exclude areas not of interest and facilitate the next steps. At this stage, the 3D polygonal models are cut with planes parallel to the axial plane. In fact, these polygonal models are only an intermediate step to isolate the abdominal tract, and the direction of the cutting plane does not affect this step. Thus, this step is aimed at reducing the portion of the aortic model under analysis, limiting it to the lower portion of the thoracic aorta and the entire abdominal area.

A raw center lumen line (CLL) is obtained from the 3D segmentation of the aortic lumen using a skeletonization algorithm [34]. This type of analysis is performed directly on the aortic lumen because it is typically more regular than the full aortic model, which also includes the thrombus (if present). Thus, the resulting skeleton is more smooth than that which can be obtained from the full aortic model. The skeleton is then exploited to automatically extract the end-point voxels (e.g., voxels with less than 2 neighbors) of the CLL that will be used to compute a more refined centerline.

Since the end-point voxels of the lumen and whole aorta correspond, the identified end-point voxels are used to compute the center aortic line (CAL) with the Vascular Modeling ToolKit (VMTK) [35] on the 3D model of the whole aorta. In order to extract the CAL, the vmtkcenterlines function with resampling step = 2.5 and smooth factor = 0.5 is used. The source seed is selected as the end point that has the highest *z*-coordinate, while the remaining end points are used as target seeds.

#### Automatic Identification of the Abdominal Centerline, Included Between the Renal and Iliac Arteries

**Fig. 4:**
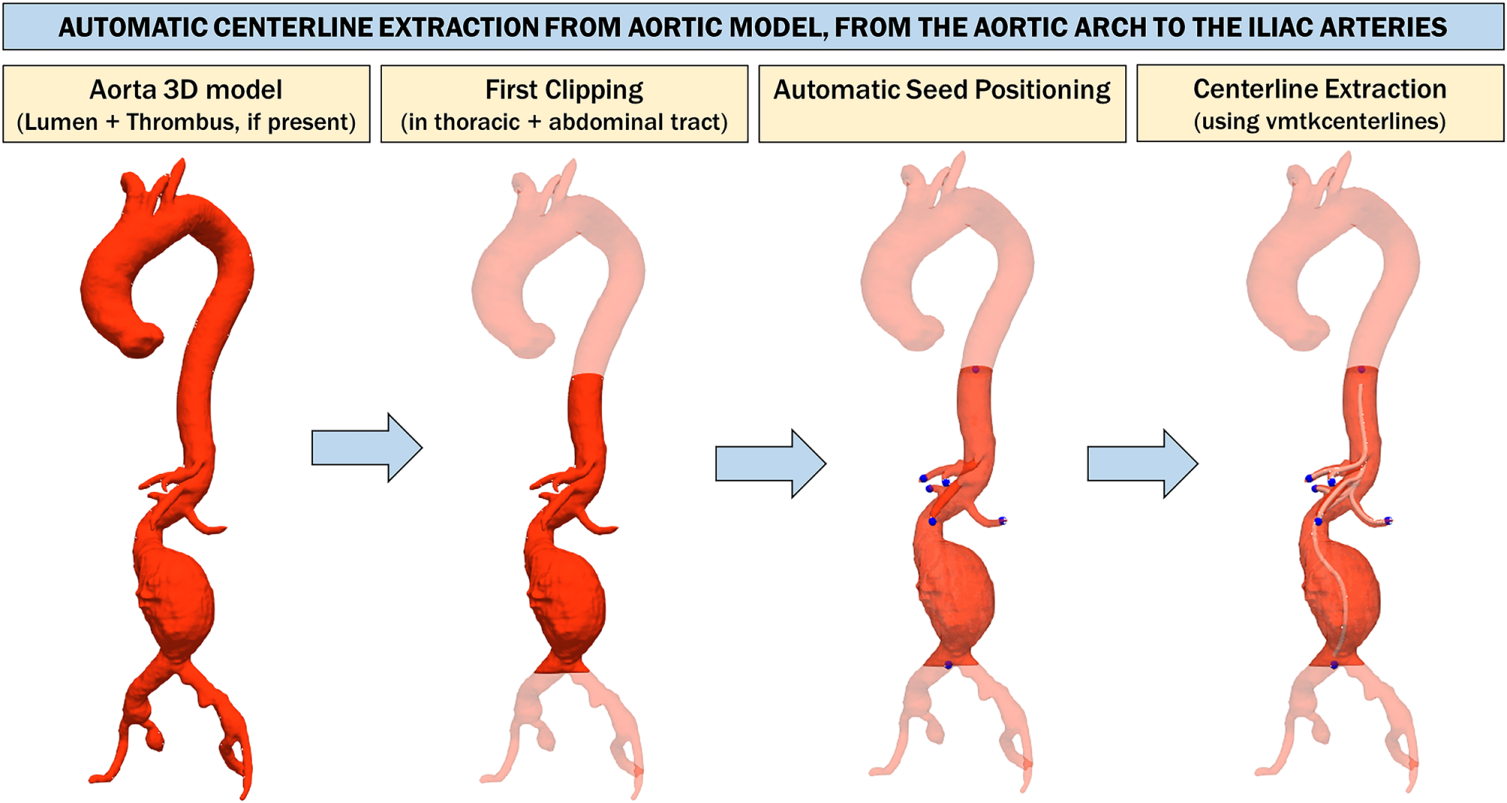
Automatic centerline extraction from aortic model, from the aortic arch to the iliac arteries. The 3D model is cut between *z*-coordinates related to the aortic arch and iliac arteries, which are identified through a process of connected component analysis performed slice by slice on the axial view. A raw centerline is obtained from this model using a skeletonization algorithm [29]. The skeleton is exploited to automatically extract centerline end-point voxels that will be used to calculate a more refined centerline with the vmtkcenterlines function of Vascular Modeling ToolKit (VMTK) [30] on the aorta 3D model

At this point, the analysis must be restricted to the abdominal tract only, clipping the 3D polygonal model between the origin of the superior renal artery and the iliac bifurcation. Thus, the *z*-coordinate in which the superior renal artery occurs must be identified (Fig. 4).

To accomplish this, the CAL is first divided and grouped along the branches using the vmtkbranchextractor function, and then, the surface is divided relative to the divided and grouped centerlines using the vmtkbranchclipper function. Next, the surface and relative centerlines, both already divided into branches, are used to calculate the bifurcation sections of the surface using the function vmtkbifurcationsections.

**Fig. 5:**
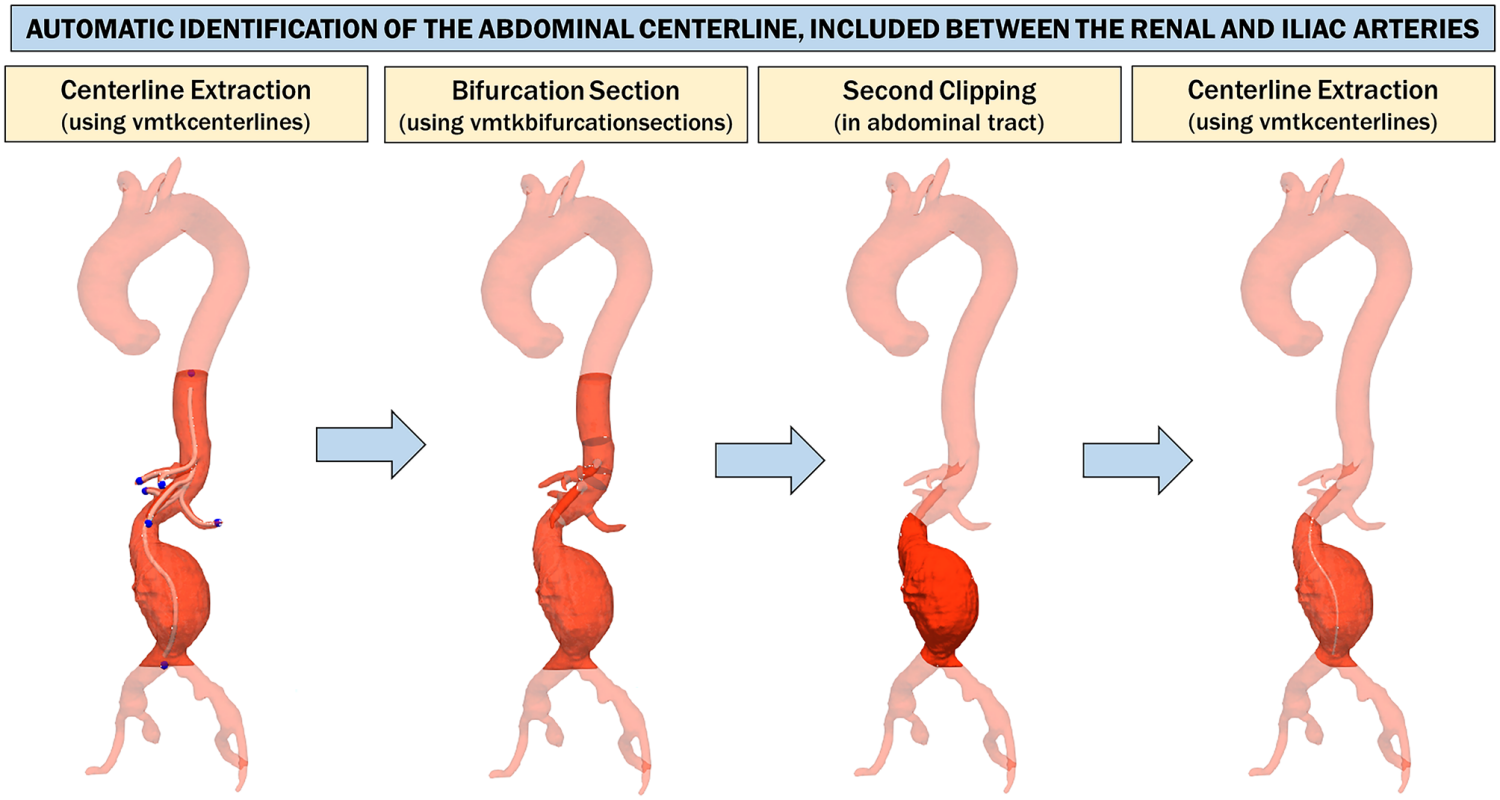
Automatic identification of the abdominal centerline, included between renal and iliac arteries. The surface and relative centerlines, both of which have already been divided into branches (using the vmtkbranchextractor and vmtkbranchclipper functions), are used to calculate the bifurcation sections of the surface using the vmtkbifurcationsections function. The last bifurcation plane extracted from the 3D model is always relative to the renal arteries. The aortic model is clipped in the abdominal area using two planes perpendicular to the centerline, centered at the height of the superior renal artery and at the height of the iliac bifurcation, respectively. The origins of these planes are used as the source seed and target seed, respectively, to recalculate the centerline of the isolated abdominal tract

As it can be noticed from Fig. 5, the last bifurcation plane extracted from the 3D model is always related to the renal arteries. The aortic model is clipped in the abdominal area using two planes that are perpendicular to the CAL: the first plane is centered at the height of the superior renal artery with the normal perpendicular to the centerline facing downward, while the second plane is centered at the height of the iliac bifurcation with normal perpendicular to the centerline facing upward. The origins of these planes are also used as source seed and target seed, respectively, to recalculate the centerline of the isolated abdominal tract.

#### Calculation of Maximum Abdominal Diameter and Comparison of Calculated Diameter with Clinical Threshold

In clinical practice, experts visually inspect CTA slices and measure the diameters in the slice where there is the largest amount of thrombus. In the proposed pipeline, the diameters are automatically extracted from the sections that are perpendicular to the centerline in the abdominal aorta. More specifically, sections perpendicular to the centerline are generated with the function vmtkcenterlinesections. The diameter of each section is computed, and the section with the largest diameter is selected among them all.

**Fig. 6:**
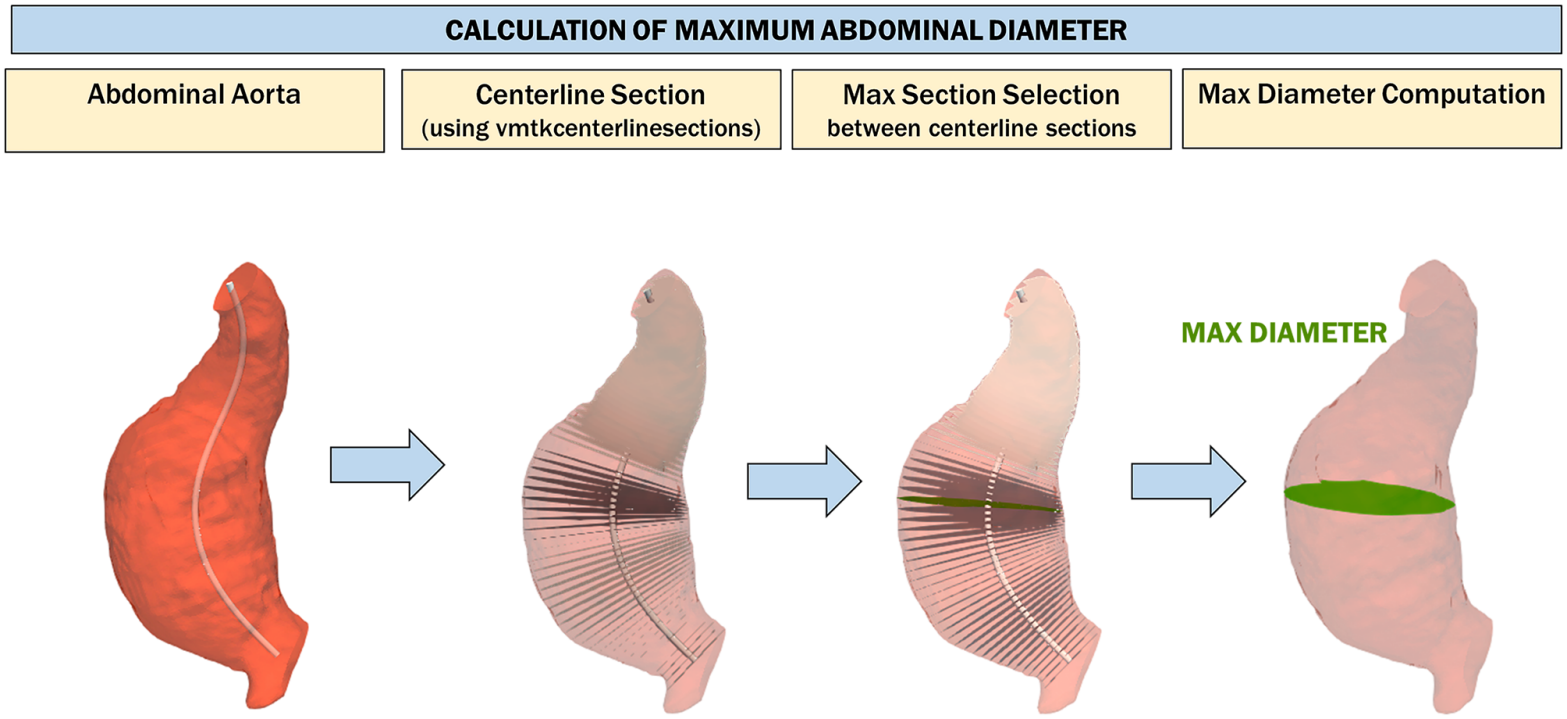
Calculation of the maximum abdominal diameter. Sections perpendicular to the centerline are generated with the function vmtkcenterlinesections. The diameter of each section is computed, and the section with the largest diameter is selected among them all

In the screening pipeline, when parietal thrombus is present, the diameters are measured from the global 3D model that includes both thrombus and lumen. Otherwise, the sections are calculated considering only the lumen surface. If the maximum diameter is greater than the selected threshold value (30 mm), the aorta is defined as aneurysmal. In addition, the developed pipeline makes it possible to assess not only the maximum aortic diameter in the abdominal district, but also the thrombus volume. In this way, the severity of the aneurysm is assessed not only locally in terms of the maximum diameter but also in terms of the global volumetric extent (Fig. 6).

## Results

### Diameter Measurement

Since the pipeline presented in this paper needed some intermediate steps before performing the analysis of the diameters in the aneurysmal sac (e.g., end-point identification from a raw skeleton and automatic abdominal centerline extraction), automatic measurements are needed to be compared to manual ones (ground truth) which are represented by the manual measurements of maximum diameter performed by the expert operator in the abdominal tract (Table 3). The absolute difference between automatic maximum diameter and ground truth is shown in Table 4. The median absolute error is 1.5 mm. It is worth considering that for healthy patients, only lumen segmentation was considered to compute the maximum diameter.

**Table 3:** Comparison of manually and automatically detected maximum diameter measurements. The main statistics of the maximum aortic diameters extracted with the proposed pipeline are shown compared with those measured by the expert

**Statistic**	**Prediction**	**GT**
Average	41.5 mm	41.3 mm
Standard deviation	18.4 mm	18.4 mm
Median	42.0 mm	42.9 mm
IQR	28.7 mm	29.0 mm
5° percentile	18.0 mm	18.3 mm
95° percentile	68.4 mm	70.6 mm

**Table 4:** Performance obtained with automatic diameter measurements. Maximum aortic diameters extracted with the proposed pipeline are compared with those measured by the expert, and performance is reported as the main statistics of the absolute error distribution of the measurements

**Statistic**	**AAA (*n*** **= 48)**	**Controls (*n*** **= 25)**	**All cases (*n*** **= 73)**
Average	2.5 mm	0.5 mm	1.8 mm
Standard Deviation	3.5 mm	0.5 mm	3.0 mm
Median	1.3 mm	0.3 mm	0.8 mm
IQR	2.3 mm	0.2 mm	1.8 mm
5° Percentile	0.1 mm	0.2 mm	0.1 mm
95° Percentile	7.7 mm	1.5 mm	6.9 mm

Our approach provides the maximum diameter of 51.2 ± 14.3 mm in patients with aneurysm and 22.0 ± 4.0 mm in patients without aneurysm.

**Fig. 7:**
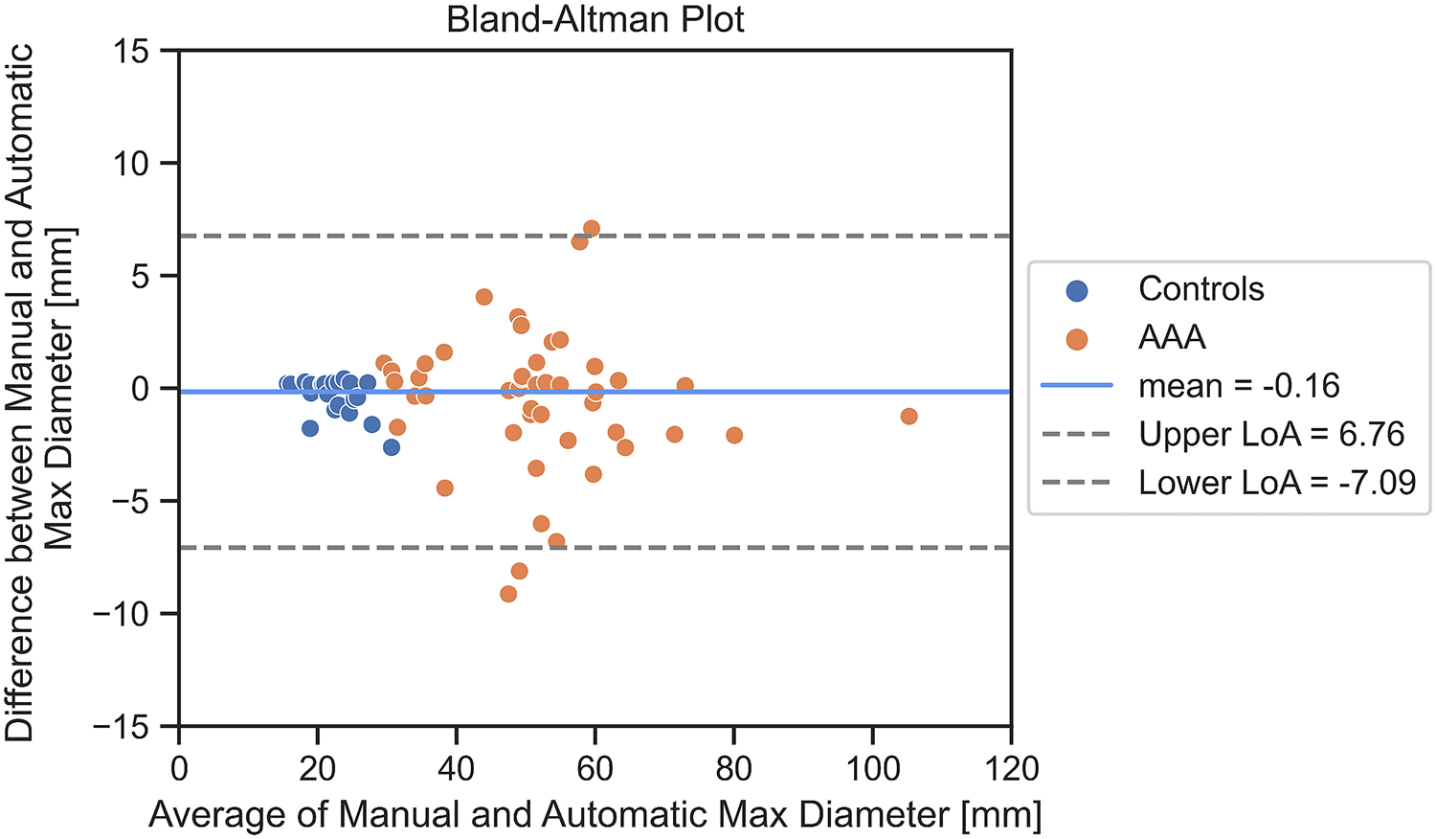
Bland–Altman plot

We also produced a Bland–Altman plot, which confirms the goodness of our results (Fig. 7). A few outliers outside the acceptable limit ([lower LoA, upper LoA]) are highlighted in the graph. From the plot, we can also see that subjects labeled as AAA by the expert show more dispersion.

#### Aneurysm Screening

**Fig. 8:**
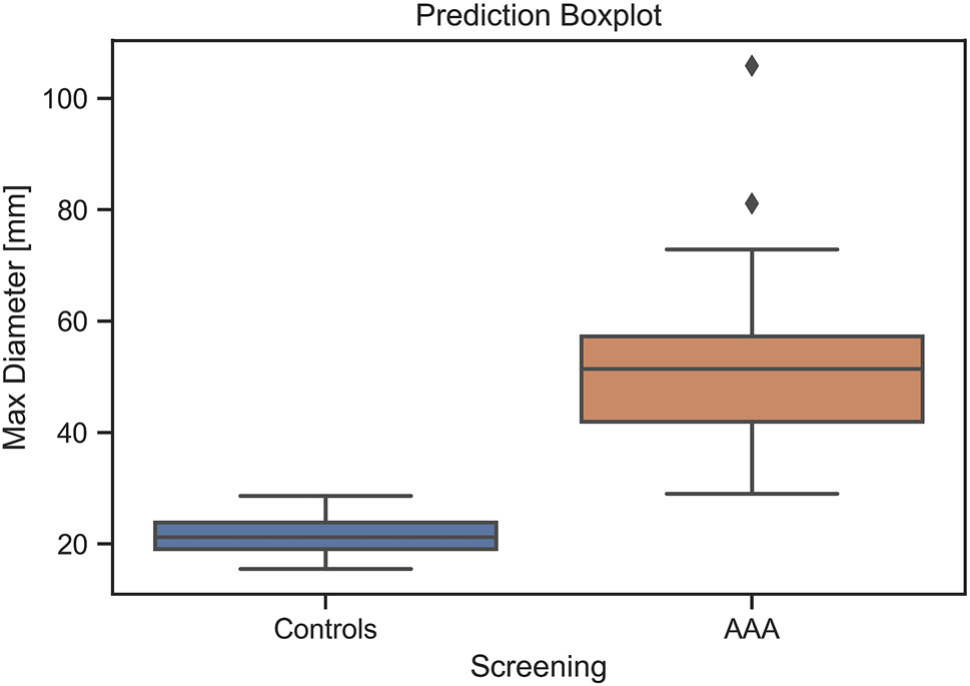
Box plot representing maximum diameters predicted in patients affected by AAA and controls

The automatic measurement pipeline is used to extract the maximum diameter in patients with and without abdominal aneurysm. As can be noticed in Fig. 8, the maximum diameter distributions obtained with the proposed pipeline are qualitatively different in the two classes, and this result is also confirmed by the *t*-test (*p* < 0.001).

**Fig. 9:**
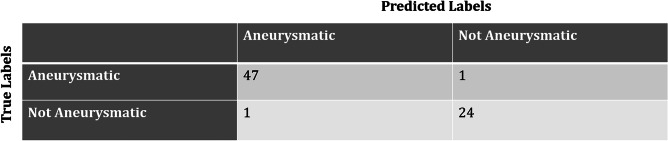
Confusion matrix

In accordance with the methodology, a threshold value of 30 mm was applied in order to determine whether the patient was aneurysmal or not. This threshold value was also applied to manual maximum diameter measurements so that a true class (e.g., AAA/controls) could be assigned to each patient. Thus, all patients with a maximum diameter measured by the expert > 30 mm were considered aneurysmal, and the others were considered healthy. Given the predicted and the expected classes, the confusion matrix was computed (Fig. 9). As it can be seen from the figure, there are two patients who are misclassified. The two patients who are misclassified by the pipeline have diameters very close to the threshold diameter of 30 mm: the patient erroneously classified as aneurysmal (AAA) has an automatic diameter of 31.9 mm vs. 29.3 mm manual diameter, while the patient misclassified as control has an automatic diameter of 29.0 mm vs. 30.1 mm manual diameter.

The resulting accuracy in AAA screening is 97% accuracy, 98% sensitivity, and 96% specificity.

In addition to measuring maximum diameter and classifying the patient as aneurysmal-non-aneurysmal, the pipeline also performed automatic measurements of thrombus volume, which in aneurysmal patients that was found to be 40 ± 49 mL, and included aneurysmal patients without thrombus and patients with maximum thrombus volume equal to 278 mL. At present, this information is purely descriptive in nature, but in future developments, the volume measurement could be coupled with the diameter measurement to perform more effective screening.

## Discussion

In this work, we proposed a DL application for automatic AAA screening from CTA images. The developed pipeline is aimed at identifying dilation starting from the aortic lumen and thrombus segmentations and at quantifying the aneurysmal sac in terms of maximum diameter.

The proposed pipeline is divided into three different parts: automatic lumen segmentation, automatic thrombus segmentation, and geometric analysis for abdominal aneurysm screening. No manual interaction is required in the whole pipeline. This pipeline is launched on a Windows computer, equipped with the NVIDIA GeForce RTX 2080 Ti graphic card with CUDA compute capability = 7.5. On average, the automatic lumen segmentation time is 25 ± 1 s per scan, the thrombus segmentation time is 63 ± 14 s per scan, and the processing time for screening is 7.12 ± 1 min.

In previous works, our group designed and validated AI-based networks to perform automatic segmentation of aortic lumen and intra-luminal thrombus [27, 28]. The aortic lumen was automatically segmented from the ascending aorta to the aortic bifurcation, including the collateral vessels that arise from the aorta, thus allowing the identification of the aortic segment located between the renal arteries and aortic bifurcation. The thrombus segmentation network, on the other hand, was trained using CTAs containing only abdominal thrombi; therefore, it could locate and segment thrombi specifically in that area. Given thrombus segmentation, a pipeline for automatic measurement of diameters in the thrombotic sac was developed. Automatic segmentation showed promising results, and the automatic maximum diameter measurement presented similar accuracy to that achievable with manual measurements.

To begin with, we have exploited the segmentation networks already proposed and validated by our group to extract the lumen and intra-luminal thrombus. Then, a centerline was automatically created in the abdominal area in order to analyze the lumen and thrombus diameters in the area of interest.

Building on previous work, a dedicated tool for automatic detection of aneurysmal dilation was implemented. Seventy-three patients were evaluated, and the AAA was detected in all cases in which the aortic diameter was ≥ 30 mm according to the reference diameter. Recently, a maximum wall-to-wall aortic diameter measurement has been proposed by some groups [20]. Specifically, an AI network has been trained to identify the maximum diameter of the aorta with no distinction between thrombus and lumen [22]. This network has allowed us to accurately measure the maximum diameter of the aneurysm, with a precision comparable to that obtained from manual measurements. Our data confirms that the AI can be used to accurately measure the aneurysmal diameter. Indeed, our DSC were 0.89 and 0.93 for lumen and thrombus segmentation, respectively [27, 28]. These results are in line with what is observed by other authors, in particular by Caradu et al. who have reported a performance of the maximum aneurysm diameter evaluation of 0.95, without distinguishing between lumen and thrombus [23].

The adoption of a 2.5D approach enables us to overcome the limitations of single 2D networks, which do not take spatial coherence on the *z*-axis into consideration, and 3D networks, which are computationally and data demanding. In particular, Golla et al. and Hwang et al. have recently developed and tested 3D classification models with several CNN architectures (AlexNet, VGG-16, resNet) through which, starting from the volume of the abdominal aorta extracted from CTAs (input), the classification of patients into aneurysmal or not-aneurysmal (output) is done [24, 36]. However, they do not perform any segmentation of either lumen or thrombus and report it as being a limitation of the study. In our approach, the DL is only used to obtain 3D volumes of lumen and thrombus, while the real screening is based on geometrical considerations (cutoff diameter 30 mm).

The segmentation of the thrombus is crucial because it allows us to distinguish between a healthy aorta and a pathological area. This aspect is pivotal for classifying the extent of the pathology but also for choosing the proper interventional approach (surgical or endovascular). For this reason, relying only on the assessment of the overall aortic diameter may not be optimal [37]. The remodeling of the thrombus inside the aneurysm sac can also be used to evaluate the evolution of the aneurysm sac until its rupture [38]. In fact, remodeling of the aorta is one of the main parameters to be considered after EVAR. Indeed, aneurysm shrinkage is considered one of the most relevant parameters for success after EVAR [38–40]. In this work, automatic thrombus and lumen segmentations are combined to assess the maximum diameter in the abdominal tract. In addition to the information on the maximum diameter, information on the volumetric extent of the thrombus is provided. Currently, it is used to have a description of the thrombotic extent in aneurysmal patients. In future works with a larger dataset, it will be possible to perform aneurysm screening by combining information on both the maximum diameter of the aneurysm and the volumetric extent of the thrombus. This way, by taking volumetric information into account, the method would be less prone to potential errors in the evaluation of the AAA maximum section.

### Limitations

There are few limitations in our study. The limited number of patients analyzed may represent a possible constraint. However, the total number of patients validated by the algorithm is consistent with the main works published in the literature. In addition, the adoption of CTAs that originate from different institutes and different CT machines allows the pipeline to be tested on images with different characteristics and to assess pipeline robustness.

Despite the manual diameter measurements being blinded to the automatic ones, another limitation is that performing the manual diameter measurements by one person can be a source of bias. To further validate the pipeline, inter-observer and intra-observer variability of the manual measurement should be performed.

Finally, the time required for the AAA analysis phase is quite high and could be optimized in order to speed up screening.

### Future Developments

As already discussed in the reported results, screening is highly dependent on automatic lumen and thrombus segmentation. For this reason, in future studies, it might be useful to retrain both segmentation models to make them, not only as robust as possible, but also able to generalize the different aortic morphologies and anatomies. Moreover, by retraining the thrombus segmentation model, it will be possible to include different types of thrombus (e.g., not only abdominal, but also thoracic and iliac) in the training set.

In this work, we developed an automated pipeline for identification of aneurysms in the abdominal area. In future developments, the same pipeline could be extended to identify aneurysms in the thoracic and iliac areas. In addition, the automatic segmentation does not consider the presence of calcifications. Since we evaluated the impact of thrombosis as more clinically relevant and more technically complex to achieve compared to calcifications, we prioritized the automatic segmentation of the thrombus. And indeed, aortic wall calcifications are more easily differentiated from nearby regions, and therefore, the implementation of the automatic network should be conceptually easier. Future studies should plan to complete the automatic segmentation regarding the segmentation of calcifications.

## Conclusions

DL applied to AAA screening represents an innovative method of study, with different applications that could have a clinical impact and help vascular surgeons in therapeutic choices and postoperative follow-up.
